# Local raster image correlation spectroscopy generates high-resolution intracellular diffusion maps

**DOI:** 10.1038/s42003-017-0010-6

**Published:** 2018-02-08

**Authors:** Lorenzo Scipioni, Melody Di Bona, Giuseppe Vicidomini, Alberto Diaspro, Luca Lanzanò

**Affiliations:** 1 Nanoscopy, Nanophysics, Istituto Italiano di Tecnologia, via Morego 30, 16163 Genoa, Italy; 2 Department of Informatics, Bioengineering, Robotics and Systems Engineering, University of Genoa, Via All’Opera Pia, 13, 16145 Genoa, Italy; 3 Department of Physics, University of Genoa, via Dodecaneso 33, 16146 Genoa, Italy; 4 Molecular Microscopy and Spectroscopy, Nanophysics, Istituto Italiano di Tecnologia, via Morego 30, 16163 Genoa, Italy; 5 Nikon Imaging Center, Istituto Italiano di Tecnologia, via Morego 30, 16163 Genoa, Italy

## Abstract

Raster image correlation spectroscopy (RICS) is a powerful method for measuring molecular diffusion in live cells directly from images acquired on a laser scanning microscope. However, RICS only provides single average diffusion coefficients from regions with a lateral size on the order of few micrometers, which means that its spatial resolution is mainly limited to the cellular level. Here we introduce the local RICS (L-RICS), an easy-to-use tool that generates high resolution maps of diffusion coefficients from images acquired on a laser scanning microscope. As an application we show diffusion maps of a green fluorescent protein (GFP) within the nucleus and within the nucleolus of live cells at an effective spatial resolution of 500 nm. We find not only that diffusion in the nucleolus is slowed down compared to diffusion in the nucleoplasm, but also that diffusion in the nucleolus is highly heterogeneous.

## Abstract (web summary)

Lorenzo Scipioni et al. present Local Raster Image Correlation Spectroscopy (L-RICS), a method for generating sub-micrometer diffusion maps. They apply L-RICS to GFP in live cells and find that diffusion coefficients differ between the nucleus and nucleolus and are highly heterogeneous within compartments.

## Introduction

The characterization of the molecular dynamics within a cell can provide important information about cellular structure and functions. More specifically, the study of protein dynamics in the nucleus can be relevant for understanding many nuclear processes and for characterizing the complex nuclear architecture^1–3^. For instance, by using biologically inert macromolecules and measuring their mobility, it is possible to gather insights about the chromatin architecture at a scale comparable to the size of the macromolecules^3–5^. For these reasons many techniques have been applied over the years for studying protein diffusion in the nuclear environment^6–11^. Among these techniques, a widely used method for measuring diffusion is single-point fluorescence correlation spectroscopy (FCS)^12^. Single-point FCS consists of shining laser light in a single diffraction-limited spot and recording the temporal fluctuations of the intensity due to the diffusion of a fluorescent probe in and out of this spot. Clearly, single-point FCS provides only local information about the molecular diffusion, but several methods have been developed for complementing the FCS data with spatial information, in order to obtain maps of the diffusion coefficient of the measured sample (and eventually correlate the maps with the intensity images).

A straightforward approach to add spatial information to FCS is performing independent single-point FCS measurements at different spatial locations. Maps of diffusion coefficients have been obtained, for instance, by interpolating scattered single-point FCS measurements^8^ or by using fast cameras in combination with light-sheet, total internal reflection or spinning disk setups^6,13–15^. Other approaches have exploited parallel fluorescence signal acquisition to get FCS data at multiple detection volumes^16–19^. In scanning FCS^20,21^ a map of diffusion coefficients is obtained along a line, even though two-dimensional maps can be obtained by performing several *x*-axis scanning FCS measurements at different *y*-axis positions^22^.

A more comprehensive framework to add spatial information to FCS is represented by the analysis of spatiotemporal correlations. This analysis is done by image correlation spectroscopy (ICS), the equivalent of FCS applied to microscopy images^23,24^. In particular, raster ICS (RICS) has been developed for measuring fast molecular diffusion by exploiting the spatiotemporal correlations contained in confocal laser scanning microscope (CLSM) images^25^. These correlations are analyzed through the spatial autocorrelation function (ACF) that depends on the diffusion of the probe and the scanning parameters. The great advantage of RICS, compared to single-point FCS, is that it is possible to measure the diffusion coefficient of a probe from an image (or a series of images) acquired on a commercial CLSM.

Unfortunately, for any given image, RICS analysis provides only an average value of the diffusion coefficient, but does not provide the spatial distribution of the diffusion properties across the image. In principle, diffusion maps can be obtained with RICS by iteratively applying the analysis to small regions of the image^26–29^. However, the size of these analyzed regions cannot be too small, since the ACF would deviate from the theoretical shape and the fitting would provide incorrect results for regions of interest smaller than 128 × 128 pixels^30^. At the typical values of pixel size used for RICS (30–50 nm) this corresponds to a spatial resolution of ∼5 μm. Clearly, this resolution value poses a limitation when spatial heterogeneity within a cell needs to be highlighted; for example, when variations of diffusion coefficient between the different regions of the nucleus need to be extracted.

In an effort to extend application of RICS to non-square regions, an approach has been introduced for computing pseudo-maps of diffusion from RICS data^31^. In this approach, the pseudo-maps are obtained by a pre-segmentation of the image based, for instance, on the intensity level, assuming that pixels with a comparable intensity level should also bear a similar value of diffusion coefficient^31^.

Recently, we have developed a method for the segmentation of ICS data sets based on the analysis of local spatial correlation functions, i.e., functions calculated on very small regions around each pixel^32^. We have shown that, in order to compare the local ACFs from all the pixels, the local ACFs can be analyzed in the frequency domain, analogous to what is done with fluorescence lifetime imaging data^33,34^. This phasor analysis of local ICS (PLICS) data can provide a fast and unbiased assessment of the heterogeneity of the correlation functions without the need of a priori information^32^.

Here, we adapt the PLICS method to work on RICS data sets for extracting local values of diffusion coefficient. We show that this local RICS (L-RICS) analysis can be used to obtain high-resolution (sub-micrometer) diffusion maps from images acquired on a CLSM. We find that, in order to reduce the noise in the map below a required level, the number of analyzed frames must be increased in a predictable way. Notably, we show that our local analysis can also be used on CLSMs equipped with non-linear scanning systems, i.e., systems in which the speed of the scanner is not constant along a line. As an application we show diffusion maps of a monomeric green fluorescent protein (GFP) in the nucleus of HeLa cells, at sub-micrometer effective spatial resolution. We show that a ∼5 min-long acquisition is sufficient to obtain a ∼5 × 5µm-sized diffusion map with an effective resolution of 500 nm and a signal-to-noise ratio sufficient to distinguish the different diffusion behavior of GFP in the nucleoplasm and in the nucleolus. By limiting the analysis to single lines, we further improve the signal-to-noise ratio of the maps, which makes it possible to resolve small differences of diffusion coefficient within the nucleolus.

## Results

### Phasor analysis of L-RICS

RICS analysis consists of computing the spatial ACF of a series of *N* images *I*_*k*_(*x*, *y*) acquired in raster-scan mode. Since the raster-scan image contains spatial and temporal information, related to the diffusion of the probe and the microscope scanning speed, the spatial correlation of the image also contains spatial and temporal information, and can be fitted to a proper model to extract the value of diffusion coefficient. The general shape of the RICS spatial ACF depends on two contributions: $G(\xi ,\eta ) = S(\xi ,\eta ) \cdot G_{{\rm diff}}(\xi ,\eta )$, where *ξ*, *η* are the spatial lags along the *x* and *y* direction, respectively, while *S*(*ξ*, *η*) is the component of the correlation function related to the laser scanning and *G*_diff_(*ξ*, *η*) is the part related to the diffusion. Considering only one spatial dimension (for instance, the *x*-axis), the theoretical formula that connects the RICS ACF to the diffusion coefficient *D* can be written as (Supplementary Note 1):

1 $$\begin{array}{rcl} G\left( \xi \right)  	=  S\left( \xi \right) \cdot G_{{\mathrm{diff}}}\left( \xi \right) \\ 	= G(0) \cdot {\mathrm{exp}}\left( { - \frac{{\left( {|\xi |K_{\mathrm{s}}} \right)^2}}{{1 + K_{\mathrm{t}}}}} \right) \cdot \left( {1 + K_{\mathrm{t}}} \right)^{ - 1} \cdot \left( {1 + \frac{{w_0^2}}{{w_z^2}}K_{\mathrm{t}}} \right)^{- 1/2}\end{array}$$

where *K*_s_ = *δx*/*w*_0_ represents the spatial sampling, namely the ratio between the pixel size *δx* and the waist *w*_0_ of the point spread function (PSF), and $K_{\mathrm{t}}{\mathrm{ = }}4D\tau /w_0^2 = \tau {\mathrm{/}}\tau _{\rm D}$ represents the temporal sampling, namely the ratio between the pixel dwell time *τ* and the diffusion time *τ*_D_ = *w*_0_^2^/4*D* of the probe. It’s worth noticing that, since the ratio between the axial and lateral waist *w*_*z*_/*w*_*0*_ is a constant that depends on the PSF of the microscope (typically *w*_*z*_/*w*_*0*_∼3 for a confocal PSF), the shape of *G*(*ξ*) depends only on the sampling constants *K*_s_ and *K*_t_.

Now, for every pixel (*i*, *j*), we define a local ACF *G*^*ij*^_*m*_(*ξ*, *η*):

2 $$G_m^{ij}\left( {\xi ,\eta } \right){\mathrm{ = }}\frac{1}{N}\mathop {\sum}\limits_{k = 1}^N {\left( {\frac{{\frac{1}{{m^2}}\mathop {\sum}\limits_x^m {\mathop {\sum}\limits_y^m {I_k^{ij}\left( {x,y} \right)I_k^{ij}\left( {x + \xi ,y + \eta } \right)} } }}{{\left( {\frac{1}{{m^2}}\mathop {\sum}\limits_x^m {\mathop {\sum}\limits_y^m {I_k^{ij}\left( {x,y} \right)} } } \right)^2}} - 1} \right)} $$

**Fig. 1:**
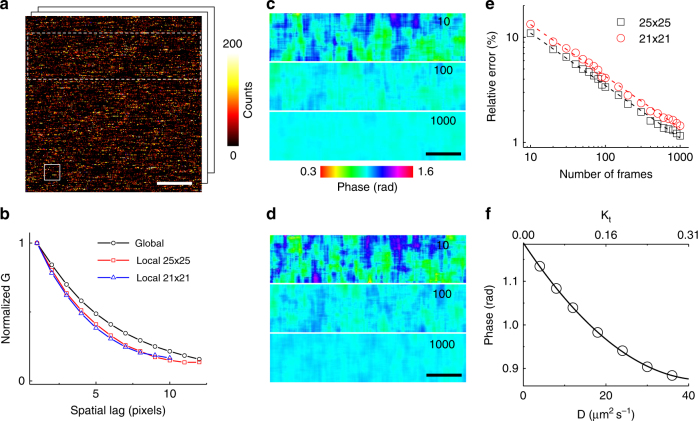
Phasor analysis of local RICS. **a** Example of one frame of a simulation with *D* = 24 µm^2^ s^−1^ and a molecular brightness of 24 kHz. The solid box shows the size of the 21 × 21 pixels mask. The white dashed box shows the region in which the analysis is performed. **b** Spatial ACF of the data set shown in **a** computed on the whole image area (black circles) and on local masks of size 25 × 25 pixels (red squares) and 21 × 21 pixels (blue triangles), respectively. **c**,**d** Phase maps obtained from analysis with L-RICS masks 25 × 25 pixels (**c**) and 21 × 21 pixels (**d**), respectively. The numbers indicate the number of frames used for the analysis. **e** Relative phase error as a function of the number of frames for a 25 × 25 pixels mask (black squares) and a 21 × 21 pixels mask (red circles). The dashed lines are power-law fits to the data with exponent *α* = −0.50 ± 0.01 and *α* = −0.49 ± 0.01 respectively. **f** Calibration curve for the 25 × 25 pixels mask and for *K*_s_ = 1/8 as a function of *D* and *K*_t_. Scale bar is 1 μm

Where *N* is the total number of frames and *I*^*ij*^_*k*_(*x*, *y*) indicates a sub-image of size *m* × *m* centered on pixel (*i*, *j*), hereafter referred to as “L-RICS mask” or simply “mask”. This local ACF can be very different from the global ACF calculated on the entire image (Fig. 1a, b) and cannot be described by the theoretical expression Eq. (1). Because of this deformation effect, due to the fact that fluctuations are sampled in a very short interval^22,30,32^, the shape of each local ACF depends also on the size of the mask. In order to analyze the local ACFs from all the pixels, we consider the 1D local ACF *G*^*ij*^_*m*_(*ξ*) (see Methods) and use the phasor approach to calculate *g*(*i*, *j*) and *s*(*i*, *j*):

3a $$g\left( {i,j} \right){\mathrm{ = }}\frac{{\mathop {\int }\nolimits_0^L G_m^{ij}\left( \xi \right){\mathrm{cos}}\left( {\frac{{2\pi \xi }}{L}} \right)d\xi }}{{\mathop {\int }\nolimits_0^L G_m^{ij}\left( \xi \right)d\xi }}$$

3b $$s\left( {i,j} \right){\mathrm{ = }}\frac{{\mathop {\int }\nolimits_0^L G_m^{ij}\left( \xi \right)\sin \left( {\frac{{2\pi \xi }}{L}} \right)d\xi }}{{\mathop {\int }\nolimits_0^L G_m^{ij}\left( \xi \right)d\xi }}$$

and calculate the phase parameter *ϕ*(*i*, *j*) (see Methods), which encodes offset-independent information about the shape of the local ACFs^32^. Here, *L* represents the number of points in which the function *G*^*ij*^_*m*_(*ξ*) is sampled, for instance for an odd-sized mask of size *m*, *L *= (*m* + 1)/2. As a result, for a given RICS data set, we get a phase map that contains spatial information on the diffusion properties (Fig. 1c, d). Smaller phase values correspond to local ACFs which decay more rapidly to zero (faster diffusion), whereas larger phase values correspond to local ACFs which decay less rapidly to zero (slower diffusion).

The noise of this phase map depends on the size of the mask and on the number of frames used for averaging (Fig. 1c–e). As expected, the error on the phase parameter follows the same general trend derived for FCS^35,36^, i.e., $\sigma _\phi \propto 1/\sqrt {t_{{\rm acq}}} \propto 1/\sqrt N$, where *t*_acq_ is the time needed to acquire *N* frames. For a given number of averaged frames, the error on the phase is larger for a smaller size of the mask (Fig. 1c–e). We calculated from simulations the number of frames required to reach a given precision in the determination of the phase. For instance, for the 21 × 21 pixels mask, at the conditions of the simulation (brightness ∼24 kHz), we can see that the error on the phase decreases from ∼15 to ∼4% by increasing the number of frames from 10 to 100. These numbers can be used to estimate the expected error in the phase for a given experiment, provided that the brightness and the acquisition parameters set for the simulation match those of the actual experiment.

Finally, in order to convert the phase map into a map of diffusion coefficients, we built a series of calibration functions, based on simulated data, for different values of *K*_s_, *K*_t_ and *m*. Indeed, for a given value of *K*_s_ and for a given value of *m*, we obtain a well-defined relationship between the phase *ϕ* and K_t_ (Fig. 1f). Provided that the dwell time is known, this relationship can be used directly to convert a value of phase *ϕ* into a value of diffusion coefficient *D* (Fig. 1f).

### L-RICS on simulated heterogeneous diffusion zones

**Fig. 2:**
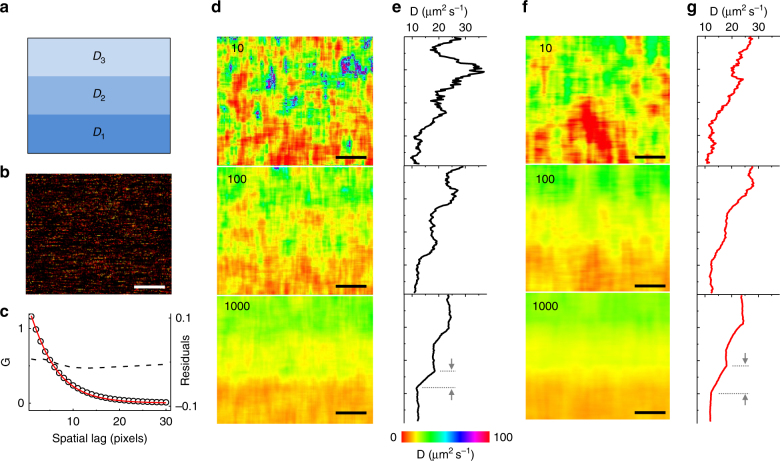
Simulations of heterogeneous diffusion zones. **a** Scheme of the diffusion coefficient distribution of the simulated data set. The values of diffusion coefficient within each zone are *D*_1_ = 12, *D*_2_ = 18 and *D*_3_ = 24 µm^2^ s^−1^, respectively. **b** Single intensity frame after time average removal. **c** *X*-axis ACF computed from the data set (black circles) along with single component fitting (solid red line) and residuals (black dashed line). **d** L-RICS diffusion maps computed with a 25 × 25 pixels mask and averaging over a different number of frames. **e** Line profiles obtained averaging each map along the *x*-axis. **f** L-RICS diffusion maps computed with a 41 × 41 pixels mask and averaging over a different number of frames. **g** Line profiles obtained averaging each map along the *x*-axis. The numbers indicate the number of frames used for the analysis. Scale bar is 1 μm

As an example of data with heterogeneous diffusion properties, we simulated a RICS acquisition on a sample consisting of three regions characterized by different diffusion coefficients (*D*_1_ = 12, *D*_2_ = 18 and *D*_3_ = 24 µm^2^ s^−1^, respectively, Fig. 2a, b). We computed the spatial ACF of the entire data set and we tried to fit the resulting function with a single component formula (Fig. 2c) along the *x*-axis. We notice that the fitting yields a satisfactory result already with a single component model (*D* = 16.8 µm^2^ s^−1^, Supplementary Fig. 1), meaning that, without a priori knowledge of the sample, it is difficult to separate the three diffusion components. Moreover, the global ACF does not convey any information about their spatial distribution. Instead, when we apply L-RICS to the same data set (Fig. 2d–g) we are able to characterize the heterogeneity of the system. Provided the statistics are robust enough, we are able to discriminate between these diffusion behaviors and we can speculate on their spatial distribution.

The spatial resolution of the diffusion map is given by the size of the mask. Comparing Fig. 2d (25 × 25 pixels mask) and Fig. 2f (41 × 41 pixels mask) we can observe that in the first case we obtain a higher spatial resolution (25 × *δx* = 500 nm) but we need to average over a larger number of frames, while in the second case the spatial resolution is lower (41 × *δx* = 820 nm) but we need considerably fewer images to reduce the noise in the diffusion map. The difference in spatial resolution can be appreciated by looking at the average *y*-axis profile at the edges of the diffusion zones (Fig. 2e, g). Notably, given that the method is entirely computational, the analysis of a given data set can be tuned, by changing the size of the mask, in such a way to reduce the noise of the map or to increase its spatial resolution.

### L-RICS of a dye in solution

**Fig. 3:**
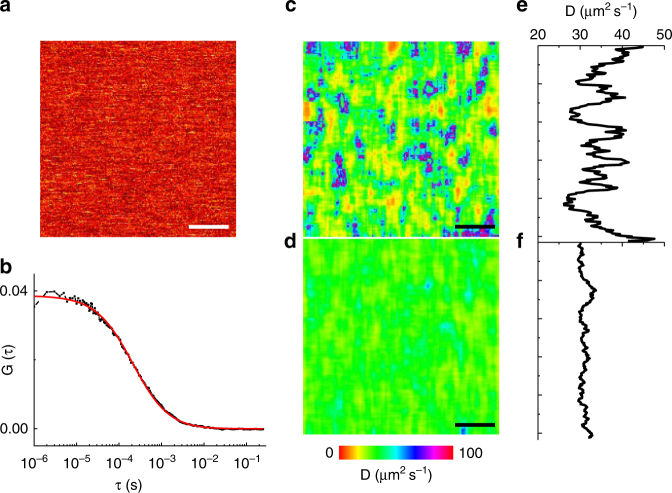
L-RICS of a dye in solution. **a** Example of one frame of acquisition of an Alexa488-labeled antibody in solution. **b** Example of a single-point FCS autocorrelation curve (black) and its fit (red). **c**, **d** Diffusion maps computed with 25 (**c**) and 250 (**d**) frames. **e**, **f** Line profiles obtained averaging each map along the *x*-axis. Scale bar is 1 μm

In order to test the algorithm on real microscopy data, we performed a RICS acquisition on a solution of an Alexa488-labeled antibody (Fig. 3a). We set the laser power to 7.2 µW entering the objective in such a way to get an apparent brightness value around 24 kHz. The sample diffusion coefficient was measured by single-point FCS (Fig. 3b), yielding a value *D*_FCS_ = 30.6 ± 1.6 µm^2^ s^−1^ (mean ± s.d., *n* = 10). The same value was obtained by standard RICS analysis (Supplementary Fig. 2) *D*_RICS_ = 30.3 ± 1.1 µm^2^ s^−1^ (mean ± s.d., *n* = 10, 250 frames). Examples of L-RICS diffusion maps obtained with a different number of frames are shown in Fig. 3c, d. The average value of *D* obtained from this analysis is *D*_L-RICS_ = 30.8 ± 2.7 µm^2^ s^−1^ (mean ± s.d., *n* = 1, *m* = 25, 250 frames) in keeping with both the result from single-point FCS and the standard RICS analysis on the same data set. For the conditions of this experiment a map with low noise can be obtained with *m* = 25 pixels (corresponding to a spatial resolution of 25 × *δx* = 500 nm) and *N* = 250 frames.

This experimental data set was compared with a simulated data set with *D*_sim_ = 30 µm^2^ s^−1^ with the same brightness and acquisition parameters and their relative phase error was plotted as a function of the number of frames (Supplementary Fig. 2), showing that both the trend and the values for the experiment match with the ones expected from the simulations, therefore validating the use of simulations as a calibration method and for estimating the error in the measurements.

### Diffusion maps of GFP in the nucleus

**Fig. 4:**
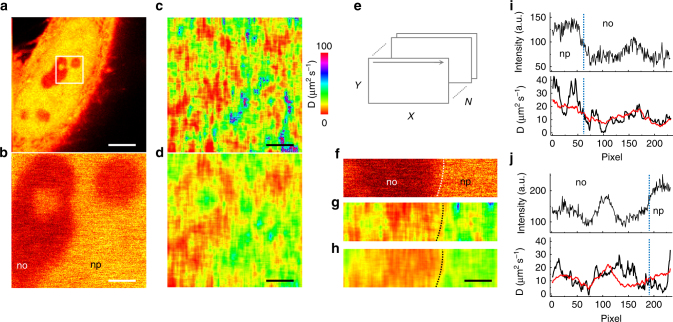
Diffusion maps of GFP in the nucleus. **a** Example of a HeLa cell expressing untagged GFP. **b** Enlarged region showing the 256 × 256 pixels area in which L-RICS was performed. Visible are portions of the nucleolus (no) and the nucleoplasm (np). **c**, **d** Diffusion maps computed with 25 (**c**) and 100 frames (**d**), corresponding to acquisition times of 1.37 and 5.46 min, respectively. **e** Schematic of the acquisition stack with dimensions *X* × *Y* × *N*. **f** Example of one frame of a L-RICS data set of dimension 64 × 256 pixels. **g**, **h** Diffusion maps obtained from **f** and computed with 100 (**g**) and 400 (**h**) frames, corresponding to 1.37 min and 5.46 min total acquisition time, respectively. **i**, **j** Examples of line maps at the nucleolus/nucleoplasm interface. Shown are the intensity profile (**i**, **j**, top) and corresponding diffusion profiles (**i**, **j**, bottom). The diffusion profiles have been computed averaging over 1000 (black line) and 10000 (red line) lines, corresponding to an acquisition time of 0.21 and 2.13 min, respectively. Scale bar in (a) is 5 μm. Scale bar in **b**–**d** and **f**–**h** is 1 μm

We then applied the L-RICS method to obtain diffusion maps of GFP within the nuclei of HeLa cells (Fig. 4). As a benchmark for the method, we tried to distinguish the different diffusion properties of GFP in the nucleoplasm and the nucleolus. To this aim, we performed the experiments in regions at the nucleolus/nucleoplasm interface, easily recognizable by the gradient in concentration of the probe in the two compartments (Fig. 4a, b). The laser power was set at 25 µW entering the objective so that the brightness of GFP was about 24 kHz. The pixel size was set to *δx* = 20 nm and the L-RICS mask size was set to *m* = 25 pixels so to obtain a spatial resolution of 500 nm. The diffusion maps obtained with 25 and 100 frames are shown in Fig. 4c, d, respectively. Since with the used acquisition parameters (*τ* = 50 µs, 256 × 256 pixels) the time needed to acquire a single frame was *t*_frame_ = 3.27 s, Fig. 4d shows that an acquisition time *t*_acq_ = 100 × 3.27 s = 5.46 min is sufficient to get an intranuclear diffusion map over an area of 5.12 × 5.12 µm^2^ and a signal-to-noise ratio sufficient to distinguish the different diffusion behavior of GFP in the nucleoplasm and the nucleolus. The observed values of diffusion coefficient in the two compartments (Supplementary Fig. 3) are in keeping with previous reports^5^. Moreover, the values of diffusion coefficients in the L-RICS map are comparable with the values of *D* obtained by performing single-point FCS measurements at various locations on the very same area and extracted by fitting the data to a model of a single diffusion component (Supplementary Fig. 4). In contrast, conventional RICS analysis performed on the whole image area does not retrieve the correct values of diffusion coefficients for the two compartments (Supplementary Fig. 5) and RICS analysis performed on a small region results in an overestimation of the diffusion coefficient (Supplementary Fig. 6).

It is worth noting that the total acquisition time *t*_acq_ is dependent mainly on the pixel dwell time *τ* and total number of acquired pixels. For *N* frames of *X* × *Y* pixels the acquisition time is *t*_acq_ ∼ *XYNτ* (Fig. 4e), assuming that line and frame retracing times are negligible. Therefore, by keeping the product *XYN* constant, it is possible to further reduce the noise on the map (increasing *N*) at the cost of decreasing the area of acquisition, without increasing the total acquisition time.

For instance, if we decrease the size of the region of interest to a 64 × 256 pixels region (Fig. 4f) we need just a fourth of the time for acquiring a diffusion map with comparable noise level and, in the same 5.46 min, we can obtain a 400-frames diffusion map in which we can more clearly distinguish the nuclear and nucleolar diffusion, and have a grasp of the sub-nucleolar heterogeneity. Pushing this methodology to its limit, we acquired single lines inside the nucleolus itself (Fig. 4g, h) with very high statistics in a short acquisition time, namely 0.21 and 2.13 min for acquiring 1000 and 10000 lines, respectively. These line maps have very good signal-to-noise ratio and can be used, in principle, to investigate the heterogeneity of diffusion within the nucleolar environment with high spatial resolution.

### Diffusion maps of GFP in the nucleolus

We performed L-RICS of line scans across nucleoli of HeLa cells co-expressing GFP and Fibrillarin-BFP2 in order to reveal small differences in the diffusion coefficient of GFP that could be associated to the inner structure of the nucleolus. Based on electron microscopy studies^37^, the nucleolus can be subdivided in three regions: the granular component (GC), which occupies the majority of the nucleolus, and the fibrillar centers (FC) surrounded by the dense fibrillar component (DFC). Fibrillarin is a protein present in the DFC and can be used as a marker to distinguish the GC from the FC/DFC regions. However, given that the FC/DFC regions below 200 nm in size, it is not possible to resolve their inner structure, at least in confocal microscopy.

**Fig. 5:**
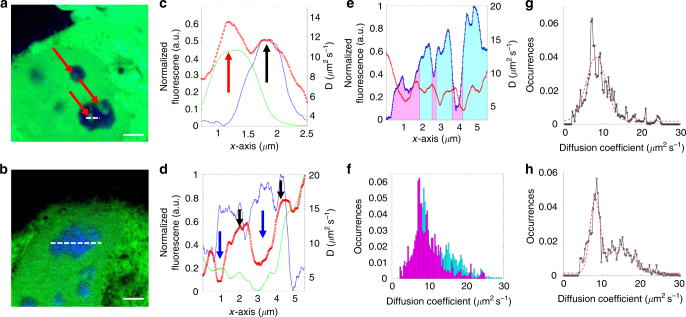
Diffusion maps in the nucleolus. **a**, **b** Representative two colors images of HeLa cells expressing untagged GFP (green) and Fibrillarin-BFP2 (blue). Red arrows indicate nucleolar vacuoles. The dashed white lines indicate the regions within the nucleolus in which line scanning was performed. **c**, **d** Line maps obtained from the acquisition indicated in **a**, **b**. The plots show the normalized intensity profiles of GFP (green) and Fibrillarin (blue) together with the diffusion profile computed with L-RICS (red circles). Black arrows indicate regions with high intensity of Fibrillarin corresponding to a high diffusion coefficient of GFP. Blue arrows indicate regions with high intensity of Fibrillarin corresponding to a low diffusion coefficient of GFP. **e** Segmentation of data in a L-RICS line map (red circles), based on the level of normalized intensity *I*_FIBR_ in the Fibrillarin channel (blue dots). Magenta and cyan areas correspond to *I*_FIBR_ < 0.5 and *I*_FIBR_ > 0.5, respectively. **f** Cumulative histograms (*n* = 15 cells) of the diffusion coefficient relative to the pixels with *I*_FIBR_ < 0.5 (magenta) and *I*_FIBR_ > 0.5 (cyan). **g**, **h** Fitting of the histograms relative to *I*_FIBR_ < 0.5 (**g**) and I_FIBR_ > 0.5 (**h**) with a single (**g**) and double (**h**) Gaussian distribution, respectively. Shown are the experimental data (black circles) and the fit (red dashed line). Data were obtained from measurements on *n* = 15 different cells. Scale bar is 2.5 μm

Representative examples of these measurements are reported on Fig. 5. As we can see (Fig. 5a, b), within the nucleolus there may be present regions in which the structure is less compacted (red arrows), resulting in a higher diffusion coefficient. These regions are characterized by a GFP signal comparable to that in the nucleoplasm and do not colocalize with the FC/DFC regions, and may be associated with nucleolar vacuoles^38,39^. Interestingly, the FC/DFC regions show a very diverse behavior when we consider their diffusion profile; for instance, we notice that they exhibit diffusion coefficients spanning over a relatively wide range, namely from less than 5 µm^2^ s^−1^ (Fig. 5d, f, blue arrows) up to more than 16 µm^2^ s^−1^ (Fig. 5b, d, f, black arrows).

In order to evaluate the spatial heterogeneity observed in the Fibrillarin signal, we segmented the diffusion maps into two groups, one containing the pixels with a high normalized Fibrillarin signal (*I*_FIBR_ > 0.5) and one containing the pixels with a low normalized Fibrillarin signal (*I*_FIBR_ < 0.5), as shown in Fig. 5e. From the analysis of a total of *n* = 15 cells, we computed the histograms corresponding to the two groups that show a clearly different distribution of diffusion coefficients (Fig. 5f–h). In particular, we find that the pixels colocalized with the Fibrillarin signal are better described by two distinct populations of diffusion coefficient values, namely one at *D*_1_ = 8 ± 1 μm^2^ s^−1^ and one at *D*_2_ = 15 ± 3 µm^2^ s^−1^. The FC/DFC regions have been linked to specific functions of the nucleolar machinery, specifically they are known to generate and accumulate transcripts within the nucleolus before their migration to the GC^37^; therefore, this difference may be due to different degrees of structural compaction that can be related to an enhanced or reduced activity for these processes, although further studies are needed to test this hypothesis.

### L-RICS on a CLSM with non-linear scanning system

Pixel dwell time is a key parameter for the correct fitting of the ACF using RICS and, in the commonly used state-of-the-art formulation, it is required to be constant within the region in which RICS is applied. This may not be true in the case of non-linear scanning systems, i.e., setups in which the scanning speed varies during the acquisition. For this reason, in the commercial systems relying on non-linear scanning, the applicability of RICS has been partly limited.

Notably, L-RICS is a local approach and only requires the scanning speed to be approximately constant over regions as small as the mask used for the analysis. Thus the method is not limited to linear scanning systems and can be used to characterize the changes of the pixel dwell time, exploiting them for correcting the diffusion map.

**Fig. 6:**
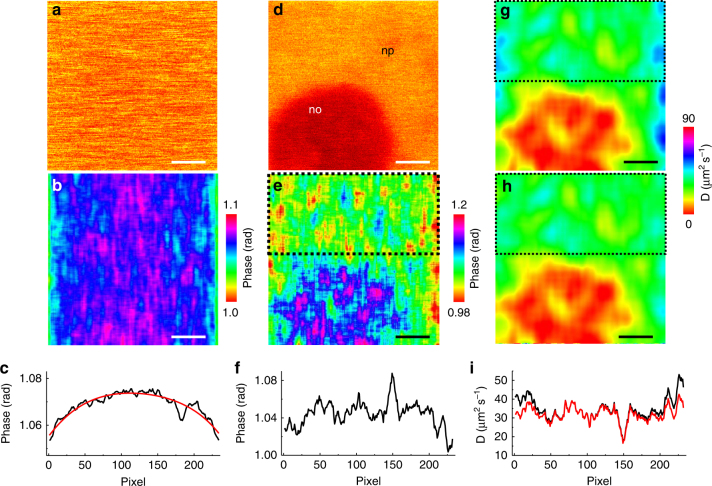
L-RICS on a non-linear scanning setup. **a**, **b** Single intensity frame (**a**) and phase map (**b**) of a solution of Alexa488-labeled antibody acquired on a non-linear scanning setup. **c** Line profile obtained averaging the phase map along the *y*-axis. The red line is a polynomial interpolation of the data. **d**, **e** Single frame (**d**) and phase map (**e**) of GFP diffusing at the nucleoplasm/nucleolus interface of a HeLa cell. **f** Line profile obtained averaging the phase map of a nucleoplasm region (black dotted box in **e**) along the *y*-axis. **g** Uncorrected diffusion map. **h** Diffusion map corrected for the non-linearity of the scanner. **i** Line profiles obtained averaging the uncorrected (black line) and corrected (red line) diffusion map of a nucleoplasm region (black dotted box in **g** and **h**). Scale bar is 1 μm

In order to test the method on data acquired using non-linear scanning, we performed experiments on a Leica SP5 microscope using 100 Hz of line-sampling frequency and we acquired 300 frames with 80% laser power (white light laser at 488 nm, corresponding to about 20 μW entering the objective) and *δx* = 20 nm. We imaged an Alexa488-labeled antibody diffusing in solution (Fig. 6a) and computed the phase map (Fig. 6b) from which we can clearly observe a gradient of scanning speed. Since the heterogeneity is present only along the *x* direction, we averaged the map along *y* in order to obtain a more statistically robust profile, and interpolated it with a polynomial function (Fig. 6c). By scaling this profile to our calibration function (Fig. 1f), we could retrieve a distribution of the *K*_t_ parameter along the *x*-axis that, in turn, can be scaled to a distribution of dwell times by knowing the diffusion coefficient of the probe (*D* = 30.6 µm^2^ s^−1^). We found that, with those acquisition parameters, the dwell time along the *x*-scanning direction varied from 12 to 18 µs (Supplementary Fig. 7). For the sake of clarity, it’s worth noting that the ‘dwell-time’ *τ* that we are measuring here by L-RICS is the time lag between two consecutive pixels of a line, i.e., *τ* = *δx*/*v* where *v* is the speed of the scanner, and not the time spent to integrate the signal at each pixel. In fact, the intensity image is not affected by the dwell-time variations (Fig. 6a).

After characterization of the inhomogeneity of the scanning system, we tested if the method was able to produce, after proper correction, diffusion maps similar to those obtained on a linear scanning setup. To this aim, we performed acquisitions at the nucleolus/nucleoplasm interface of HeLa cells expressing GFP (Fig. 6d) and computed the phase map obtained by L-RICS analysis on 300 frames (Fig. 6e). This phase map encodes variations of the local correlation functions due not only to the value of the diffusion coefficient (for instance between nucleoplasm and nucleolus) but also to the value of pixel dwell time. Indeed, the phase map is deformed toward lower values at the borders of the image due to the slower scanning speed, as visible from the phase profile averaged over a nucleoplasm region (Fig. 6e, f). As a result, the diffusion map computed using a constant pixel dwell time (Fig. 6g) is biased at the borders toward higher values of diffusion coefficients (+10 to +15 µm^2^ s^−1^). Conversely, when we compute the diffusion map using the dwell time gradient obtained from previous calibration (Fig. 6c), we get a corrected map that shows no bias at the borders (Fig. 6h, i).

## Discussion

In this work, we developed and characterized a computational method for probing and quantifying the spatial heterogeneity of diffusion with sub-micron resolution, based on the analysis of images acquired on a CLSM. In terms of spatio-temporal resolution and capability to explore large areas, the L-RICS method shows intermediate features between RICS and single-point FCS. Compared to single-point FCS, L-RICS offers several advantages. First of all, L-RICS can be used to compute high-resolution diffusion maps from images acquired on readily available confocal microscopes, thus without the need of a dedicated setup for acquisition of FCS data at multiple locations on the sample^18,19^. Second, the use of the phasor approach as a fit-free method to estimate the value of diffusion constant at each pixel, results in an easy-to-use tool that does not require the fitting procedures normally employed in FCS data analysis. Finally, the way data are acquired in L-RICS (i.e., a series of consecutive images) is convenient to check for instabilities of the sample during the acquisition (e.g., movements of the whole cell), whereas, in FCS, these instabilities may affect directly the ACF but be of less obvious interpretation. On the other hand, L-RICS has a major limitation, compared to FCS: the L-RICS analysis is oversimplified (extraction of a diffusion constant value *D* at each pixel) as its main purpose is to detect spatial heterogeneities of diffusion. Thus, the L-RICS method (at least in the present formulation) does not offer the sensitivity of single-point FCS to analyze quantitatively other processes (e.g., binding, triplet reactions) occurring during diffusion. Similarly, it cannot discern between pure Brownian motion and diffusion affected by spatial confinement. Nevertheless, even in situations that should be described by more complex diffusion models, L-RICS can be used as a pre-screening assay to get a map of ‘apparent’ diffusion constant *D*, useful as a guide to perform single-point FCS at specific locations.

The L-RICS method is based on the analysis of local spatial ACFs that are calculated over small regions and are thus sensitive to the local value of diffusion coefficient. We have shown that, as expected, the local ACFs are noisier when compared to the ACF calculated over the whole image. We compensate for this by averaging the local ACFs over a larger number of frames. Notably, we have shown that, by using simulations, it is possible to obtain a priori information about the signal-to-noise ratio of the map achievable with a given experiment. In other words, if we want to obtain a diffusion map at a given spatial resolution (determined by the size of the mask and the pixel size) and noise below a given level, we will need to average the local ACFs over a minimum number of frames *N*_min_. In order to get an accurate map, the sample must be stationary over the time needed to acquire *N*_min_ frames. This may prevent application of our method to subcellular regions and/or structures that are relatively mobile during the acquisition and for which other methods, based on labeling and/or tracking the organelle of interest, appear to be more suitable^31,40,41^. Another factor that can limit the duration of the experiments is represented by photobleaching (Supplementary Fig. 8).

Nevertheless, a reduction of the whole acquisition time can always be obtained by reducing the size of the scanned image (Fig. 4e). In this respect, we have demonstrated that the method can also be applied to line scanning to produce one-dimensional diffusion maps of very low noise in a relatively short time. It’s worth noting that these line-RICS maps are different from those obtainable by scanning FCS. In scanning FCS the temporal resolution is given by the line time *τ*_*l*_, typically in the order of ∼1 ms, providing accurate measurement of diffusion coefficients only below *D*_max_ ∼ *w*^2^/4*τ*_*l*_^21^. Even if this temporal resolution is generally sufficient to study diffusion of intracellular proteins in confocal or 2-photon FCS (*w* ∼ 0.2 μm, *D*_max_ ∼ 10 μm^2^ s^−1^), it might be limiting in super-resolution stimulated emission depletion (STED)–FCS, where the observation volume can be significantly smaller^42,43^. In contrast, the temporal resolution of our line-RICS maps is not limited by the line scan time and is basically the same as in single-point FCS, making the method compatible, at least in principle, with super-resolution, such as STED microscopy. The combination with recently developed methods to perform efficient STED-FCS in 3D^44,45^ can eventually result in line-maps of diffusion coefficient measured at different observation volumes, which might be useful to detect anomalies of motion similarly to what has been done in lipid membranes^46^. In particular, the coupling of L-RICS with STED–fluorescence lifetime correlation spectroscopy (FLCS) methods^45,47^ would result in the possibility of distinguishing diffusion modalities different from pure diffusion, which as of now is a limiting factor of the L-RICS approach.

A distinctive feature of our local analysis is that it can reveal the potential inhomogeneity in the scanning speed of a given setup and correct for it, therefore allowing the use of L-RICS also in systems in which the application of RICS is less straightforward. We believe that the generality and ease of implementation of this method can be of great help in the study of diffusion in the cellular environment; given that the cell is an intrinsically heterogeneous system, in both space and time, averaging over extensive areas can result in the loss of information about the local dynamics, ultimately providing an inaccurate or biased value.

In order to show that L-RICS can provide a way to overcome this limitation, we measured the diffusion map of GFP in the intranuclear space of HeLa cells at different levels of signal-to-noise ratio, demonstrating that the GFP diffusion in the nucleus is highly heterogeneous and exhibits diffusion coefficients that span over an order of magnitude. In particular, by exploiting the high level of signal-to-noise ratio achievable by the line-RICS maps, we could also detect the heterogeneity of GFP diffusion within the nucleolus. We simultaneously measured the diffusion coefficient of FC/DFC regions and nucleolar vacuoles, reporting differences in diffusion coefficient that are probably related to variations of structural compaction within the nucleolus. Emerging models of nuclear organization describe heterochromatin domains and nucleolar subcompartments as liquid phases with distinct biophysical properties^48,49^. We believe that our method can be a valuable tool to characterize the heterogeneous diffusion properties associated to these compartments.

## Methods

### Local-RICS algorithm, simulations and data analysis

The entire computational part was performed in MatLab (Mathworks).

For every pixel (*i*, *j*) of an image *I*_*k*_(*x*, *y*) the algorithm computes the local ACF *G*^*ij*^_*m*_(*ξ*, *η*) of a sub-image of size *m* × *m* around that pixel. The 2D-ACF is calculated by a 2D-FFT (fast fourier transform) on the sub-image and then averaged over *N* frames in order to obtain a more robust ACF. Each 2D-ACF is transformed into a 1D–ACF, by taking into account only the component along the *x* direction, *G*^*ij*^_*m*_ (*ξ*) = *G*^*ij*^_*m*_ (*ξ*, 0). Since in the conditions of all our experiments the time lag between two lines is too large to detect any correlation, this is the only meaningful component of the 2D-ACF. The phasor variables *g*(*i*, *j*) and *s*(*i*, *j*), defined by Eq. (3a, b), are calculated by performing a 1D-FFT on the function *G*^*ij*^_*m*_(*ξ*). The phase coordinate *ϕ*(*i*, *j*) is obtained by the simple operation:

4 $$\phi (i,j){\mathrm{ = }}{\rm tan}^{ - 1}\left(\frac{{s(i,j)}}{{g(i,j)}}\right)$$

Before performing the 1D-FFT, we always assign to the zero lag point of the ACF, which contains the white noise autocorrelation, the same value as the first lag.

In order to extract a quantitative relationship between the phase value *ϕ*(*i*, *j*) and the local value of diffusion coefficient *D*(*i*, *j*), we performed simulations of fluorescent molecules freely diffusing with a certain diffusion constant *D* and raster-scan acquisition parameters described by the sampling constants *K*_s_ and *K*_t_ (Fig. 1a).

All simulated data were generated in SimFCS (available at www.lfd.uci.edu/globals/). Several RICS data sets of 2500 or 500 molecules undergoing 3D diffusion were simulated with *w*_0_ = 0.16 µm, *w*_z_ = 3w_0_, pixel dwell time *τ* = 50 µs, pixel size *δx* = *δy* = 20 nm, molecular brightness *B* = 24 kHz or 3 MHz and several diffusion coefficients and number of frames (Supplementary Movies 1–8). For calibration, we obtained the reference curves *ϕ*(*K*_t_) by simulating the data sets with different diffusion coefficients in SimFCS and by plotting the retrieved average phase value as a function of the simulated diffusion coefficient. For the acquisition parameters and the samples chosen in this work, the calibration curve was obtained by simulating diffusion coefficients in the range 4–36 µm^2^ s^−1^. By simulating several data sets with high molecular brightness (*B* = 3 MHz, 250 images) varying *K*_t_ and keeping *K*_s_ constant (*K*_s_ = 1/8), we were able to construct a calibration curve linking the phase to the diffusion coefficient (or to *K*_t_ itself) for the appropriate mask. The resulting curve was then fitted to an exponential decay, the parameters of which were stored; once the phase image *ϕ*(*i*, *j*) is computed for an experiment, it is successively inverted through the appropriate calibration parameters in order to obtain the diffusion map *D*(*i*,*j*).

For the analysis of data obtained in a non-linear scanning setup, we first computed a map of dwell time *τ*(*x*, *y*) from the phase map shown in Fig. 6b. To this aim, the phase map was averaged along the *y*-axis and fitted with a polynomial function, in order to obtain a smoother profile. This profile was then inverted to obtain the profile of *K*_t_(*x*) along the *x*-axis. The dwell time was then calculated as *τ*(*x*) = *K*_t_(*x*)*w*_0_^*2*^/4*D*. A dwell time map *τ*(*x*,*y*) was obtained by copying the dwell time profile *τ*(*x*) along the *y*-axis (Supplementary Fig. 7).

A background subtraction was performed by a moving average subtraction over time, the mean value of intensity subtracted is then added back to the image as a constant offset. For simulations, the average over the whole data set was subtracted.

The error of the phase parameter *σ*_*ϕ*_ was evaluated as the standard deviation calculated over the entire phase image.

Conventional RICS analysis was performed by computing the spatial ACF corresponding to the whole image area:

5 $$G\left( {\xi ,\eta } \right) = \frac{1}{N}\mathop {\sum}\limits_{k = 1}^N {\left( {\frac{{\left\langle {I_k\left( {x,y} \right)I_k\left( {x + \xi ,y + \eta } \right)} \right\rangle }}{{\left\langle {I_k\left( {x,y} \right)} \right\rangle ^2}} - 1} \right)} $$

and fitting the ACF to the model described by Eq. (1).

### Samples

For the measurement of a dye in solution, a goat anti-mouse antibody coupled with Alexa 488 (Life Technologies) was diluted to a concentration of 20 µg/ml in PBS.

A stable HeLa cell line expressing the protein AcGFP1 (ClonTech) was used for all the experiments^50^. The day before the experiment, freshly split cells were plated on LabTek or Ibidi 8-well chamber (glass bottom, thickness 170 ± 5 µm) and let them grow overnight.

Transfection with EBFP2-Fibrillarin-7 (gift from Michael Davidson, Addgene plasmid # 55241) was performed with Lipofectamine 2000 (Thermofisher Scientific) following manufacturer instructions.

The brightness of AcGFP1 at different laser powers (Supplementary Fig. 9) was measured using an aqueous solution of purified AcGFP1 (Clontech), prepared by diluting the protein in PBS (phosphate-buffered saline 1×, Thermo Fisher Scientific) at a final concentration of ∼100 nM. The brightness of AcGFP1 was calculated from single point FCS measurements as *B* = <*I*>/<*N*_mol_>, where <*I*> is the average intensity and <*N*_mol_> is the average number of particles in the confocal volume.

### Microscopes and experiments

The samples were imaged by a custom confocal microscope or by a Leica SP5 STED confocal microscope, both equipped with Leica 1.40 NA 100× objectives (HCX PL APO 100× 1.40/0.70 Oil, Leica Microsystems).

The custom microscope was obtained as a modification of a previous setup^51^. Briefly, the excitation at 485 nm was provided by a picosecond (<100 ps) pulsed (80 MHz) laser diode (LDH-D-C-485 Sepia, PicoQuant). Excitation at 405 nm was provided by a CW laser diode (Cube 405, Coherent). The two beams were combined using two dichroic mirrors, then deflected by two galvanometric scanning mirrors (6215HM40B, CTI-Cambridge) and directed toward the objective by the same set of scan and tube lenses as the ones used in a commercial scanning microscope (Leica TCS SP5, Leica Microsystems). The fluorescence light was collected by the same objective lens, de-scanned, passed through the dichroic mirrors, then separated in two channels (525/50 nm and 445/45) before being focused (focal length 60 mm, AC254-060-AML,Thorlabs) into fiber pigtailed single-photon avalanche diodes (PDM Series, Micro Photon Devices). All imaging operations were automated and managed by the software Imspector (Max Planck Innovation). For single-point FCS, photons were detected by a TCSPC (Time Correlated Single Photon Counting) card (SPC-830, Becker & Hickl), synchronized with the reference signal provided by the pulsed diode laser. The power of the laser beam was always measured before entering the objective. Due to losses in the objective lens, the power at the sample is actually lower by 15%.

For FCS measurements, ten data sets with 30 s acquisition time were acquired at a laser power of 9 µW. The raw data were processed for afterpulse removal using a custom FLCS algorithm^45^.

### Custom code

A version of the L-RICS algorithm running under Matlab is provided in Supplementary Software 1.

### Data availability

Simulated RICS data sets are provided as Supplementary Movies 1–8. The data that support the findings of this study are available from the corresponding author upon reasonable request.

## Electronic supplementary material

Supplementary Information

Description of Additional Supplementary Files

Supplementary Software 1

Supplementary Movie 1

Supplementary Movie 2

Supplementary Movie 3

Supplementary Movie 4

Supplementary Movie 5

Supplementary Movie 6

Supplementary Movie 7

Supplementary Movie 8
