# Supplementary material for: Local raster image correlation spectroscopy generates high-resolution intracellular diffusion maps
Source: Commun Biol. 2018 Feb 8;1:10. doi: 10.1038/s42003-017-0010-6 (PMC6053083; doi:10.1038/s42003-017-0010-6)
Supplement: Supplementary file 1 — Supplementary Information [file 42003_2017_10_MOESM1_ESM.pdf]

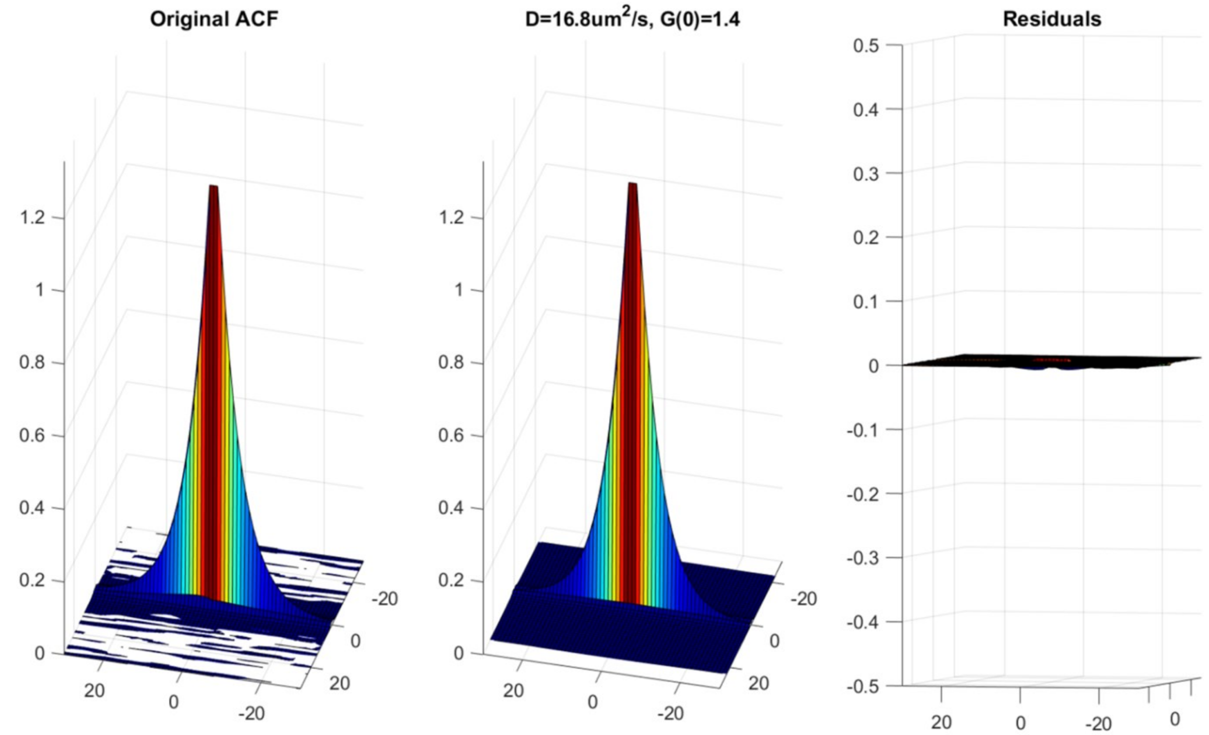

Supplementary Figure 1. RICS analysis of the heterogeneous three components simulation shown in Fig. 2 in the main text. Shown are the ACF (left), a one-component fit (center) and the residuals (right).

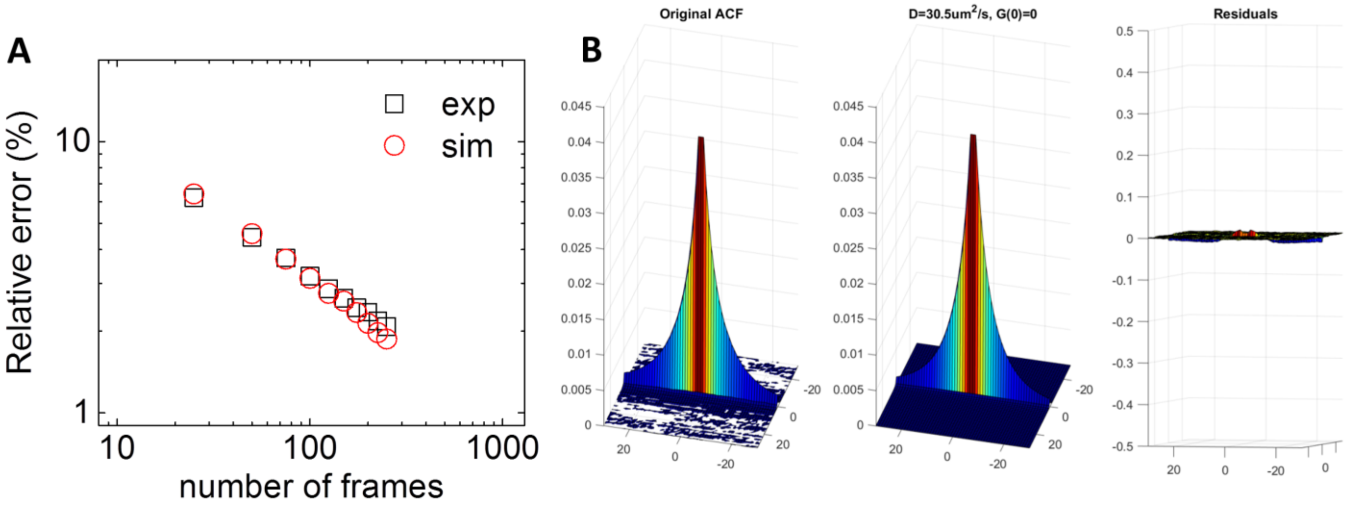

Supplementary Figure 2. (A) Relative error as a function of the number of images used for the L-RICS analysis. The plot shows the relative phase error for the dataset shown in Fig. 3 in the main text (black squares) and for data simulated using the same diffusion coefficient and brightness (red circles). (B) Conventional RICS analysis for a free dye diffusing in solution. Shown are (from left to right) the RICS ACF, a fitting to a free diffusion model and residuals. The fitting yields D_{RICS}=30.5 $\mu m^2 s^{-1}$.

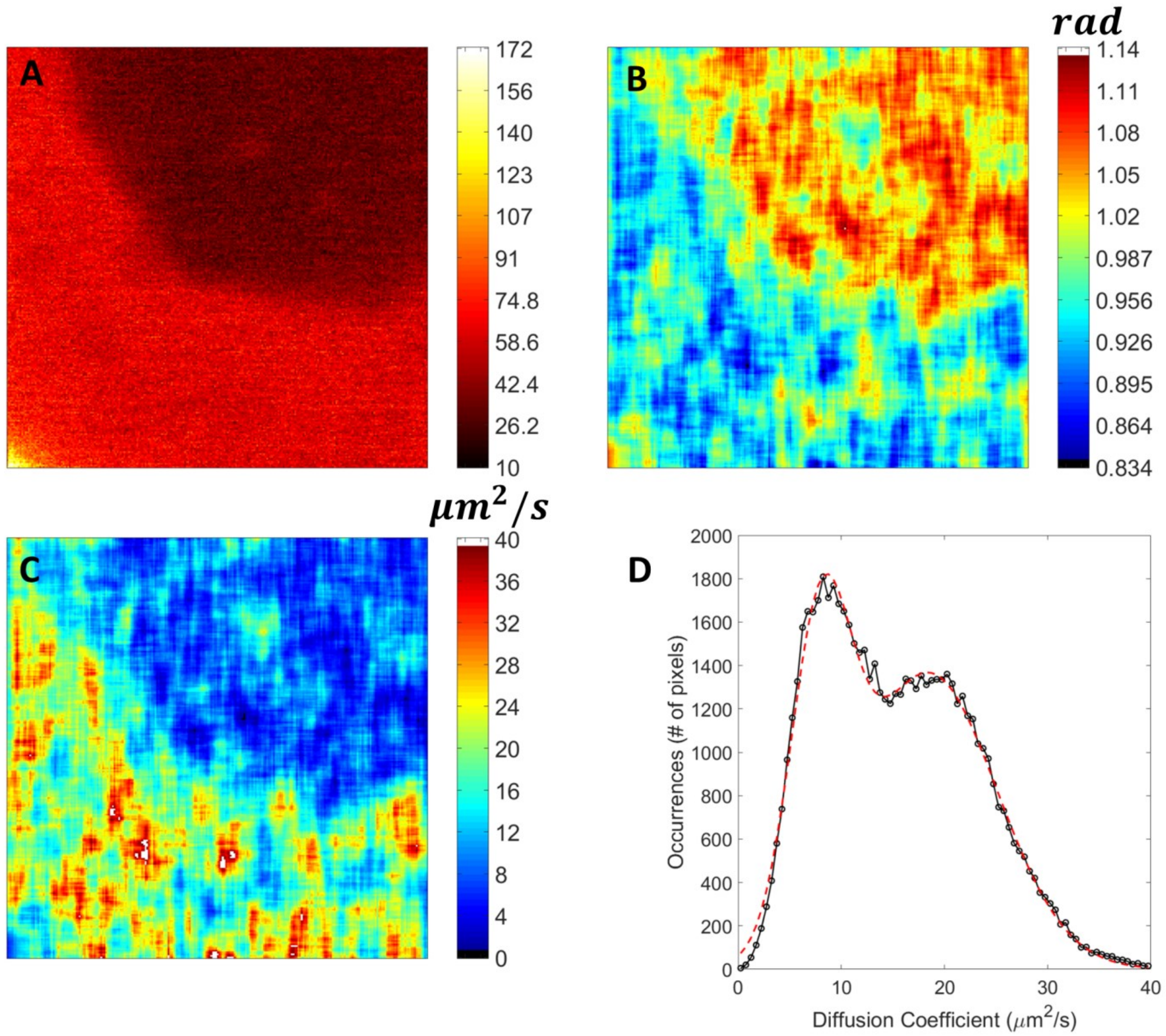

Supplementary Figure 3. Example of diffusion map of GFP in the nucleus. (A) Example of one frame of a 100-frames acquisition at the nucleoplasm/nucleolus interface. (B,C) Phase (B) and diffusion (C) map relative to the same dataset. (D) Histogram computed from the diffusion map (black circles) and two components Gaussian fitting (red dashed line). The fitting yields D_1=18.5±6.6 $\mu m^2/s$ and D_2=8.0±2.9 $\mu m^2/s$

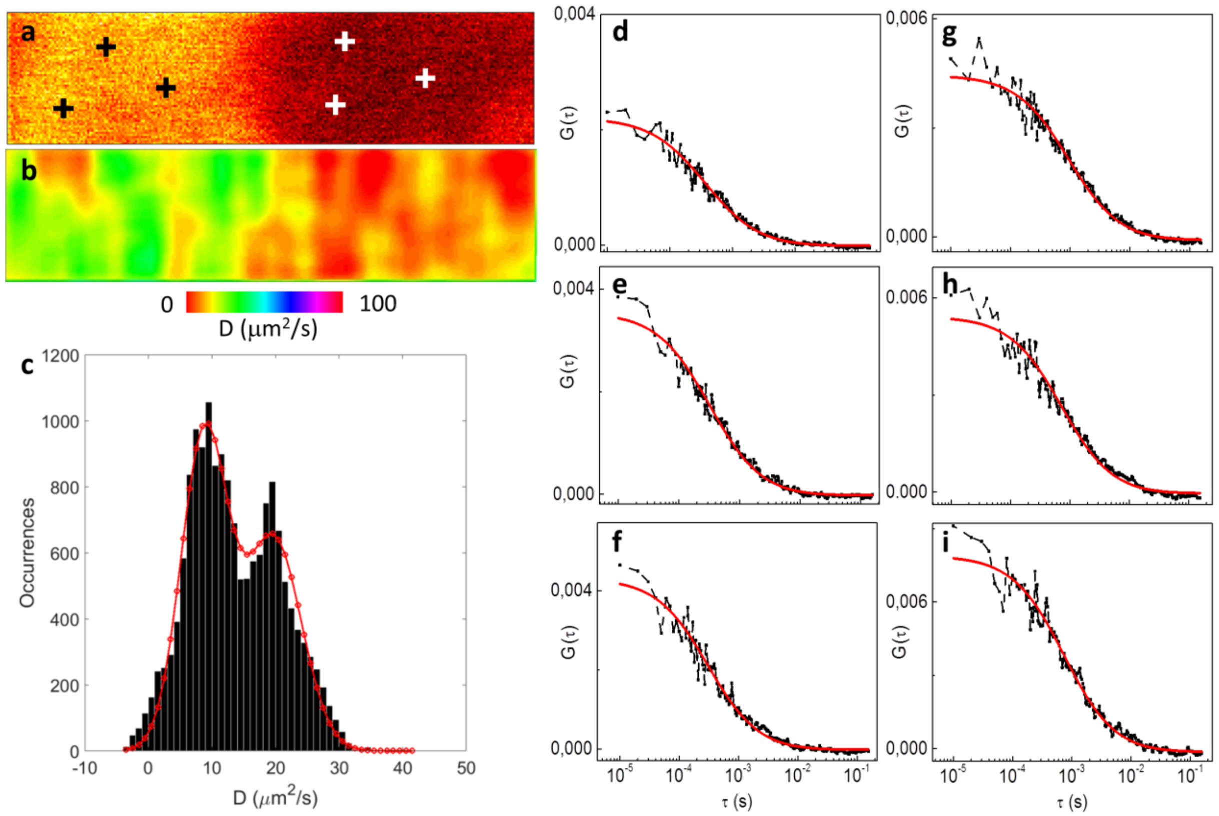

Supplementary Figure 4. Intensity image (**a**) and diffusion map (**b**) of GFP diffusing at the nucleoplasm/nucleolus interface. Histogram (**c**, black bars) and two-components fitting (**c**, red line) of the retrieved diffusion coefficients; the fitting yields D_1=8.8±3.7 $\mu m^2/s$ and D_2=19.7±4.4 $\mu m^2/s$. FCS curves (black lines) acquired in the nucleoplasm (**d**-**f**, black crosses in **a**) and nucleolus (**g**-**i**, white crosses in **a**) and one-component fitting (red lines); the fittings yield D=17, 20 and 21 $\mu m^2/s$ for the nucleoplasm and D=6.8, 9.0 and 8.6 $\mu m^2/s$ for the nucleolus, respectively.

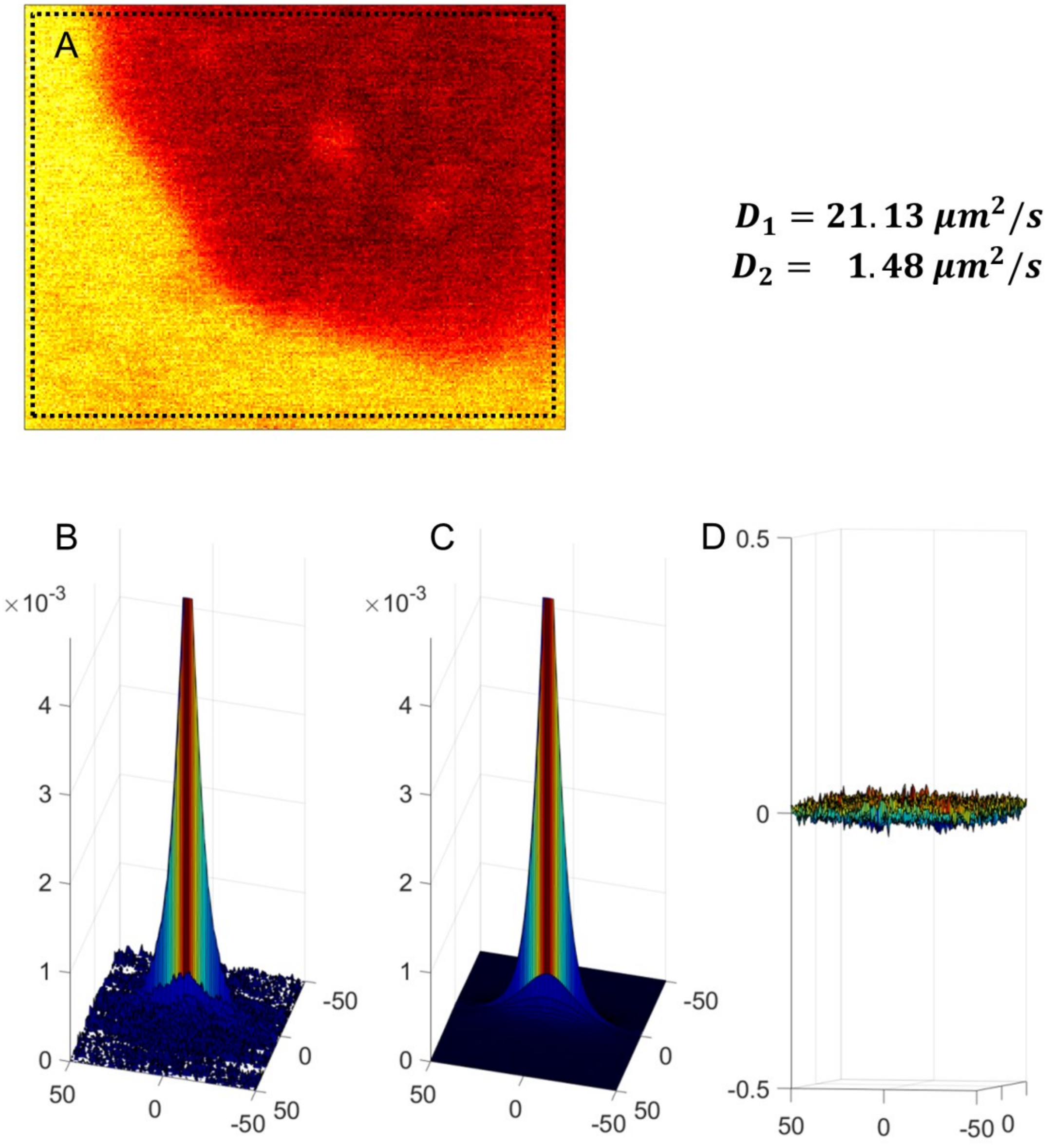

Supplementary Figure 5. (A) Example of one frame from an acquisition at the nucleoplasm/nucleolus interface. Autocorrelation function (B) calculated from the highlighted region in A (201x256 pixels), averaged over 256 frames, 2 components fit (C) and residuals (D). The fitting yields $D_1 = 21.1\ \mu m^2/s$ and $D_2 = \ 1.5\ \mu m^2/s$

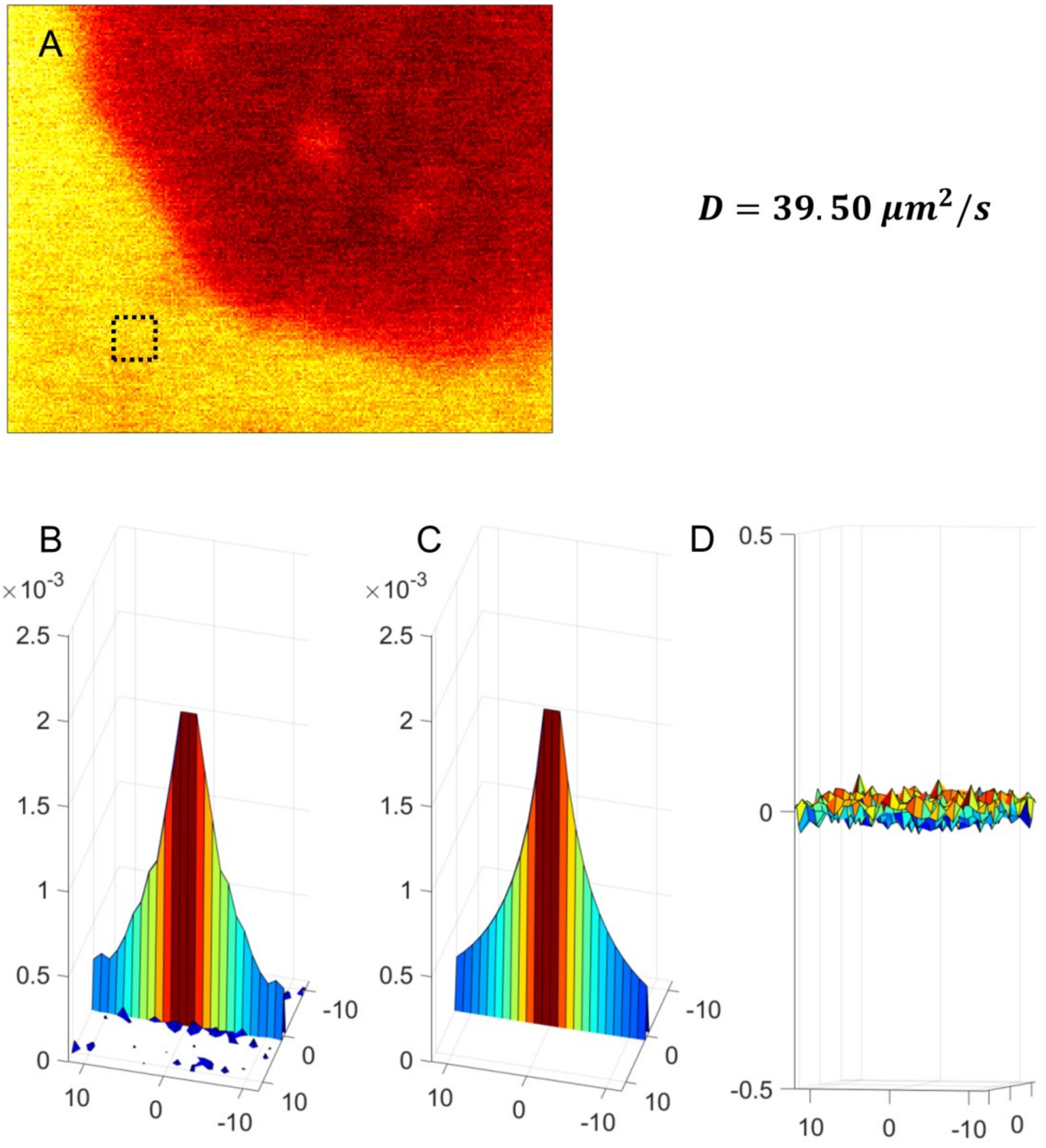

Supplementary Figure 6. (A) Example of one frame from an acquisition at the nucleoplasm/nucleolus interface. The analysis shows the RICS autocorrelation function (B) calculated from the highlighted region in A (25×25 pixels), averaged over 256 frames, a single component fit (C) and residuals (D). The fitting yields D_{RICS}=39.5 μm^2/s whereas the corresponding value recovered by L-RICS on the very same area is $D_{L\text{-}RICS}$= 16 μm^2/s.

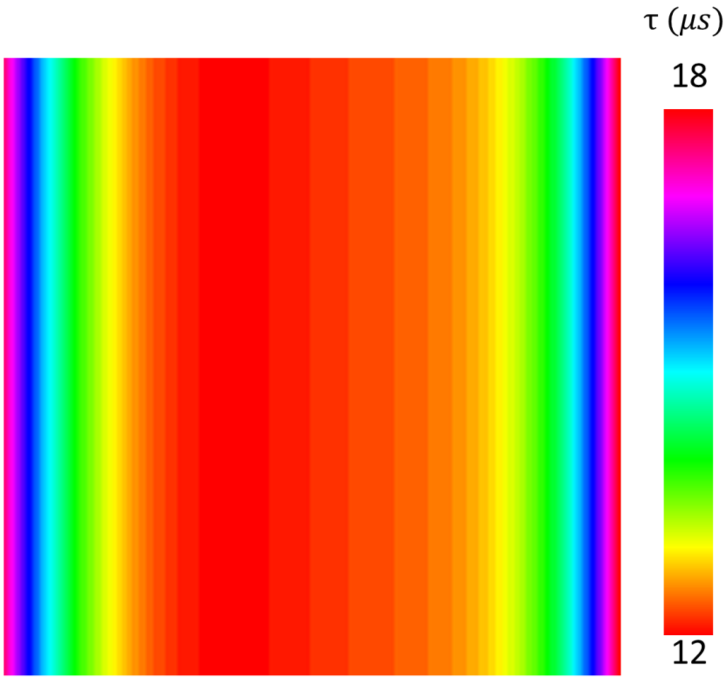

Supplementary Figure 7. Dwell time map of a non-linear scanning setup. The map has been obtained by inverting the phase profile shown in Fig.6 C in the main text.

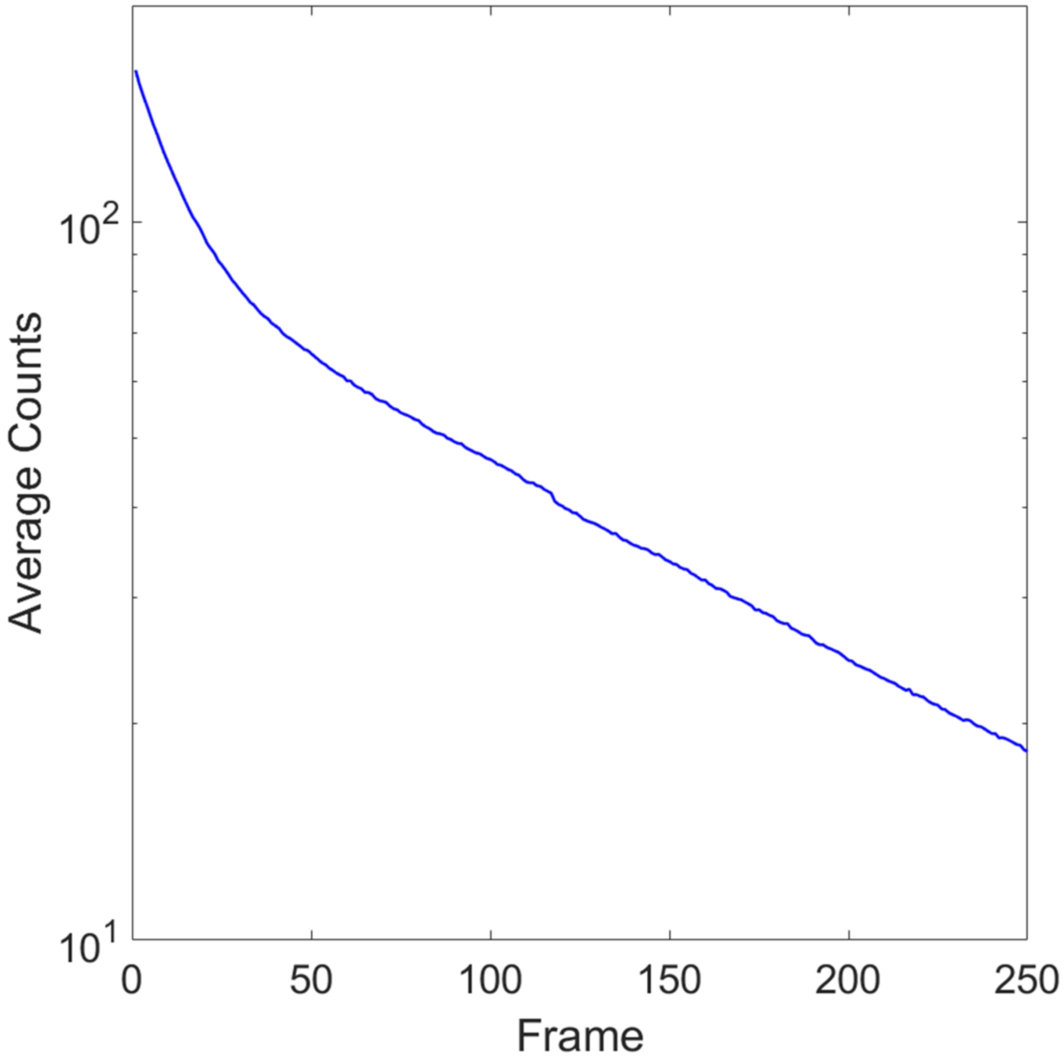

Supplementary Figure 8. Photobleaching curve relative to the dataset show in Supplementary Fig. 5 and 6, computed over the entire image.

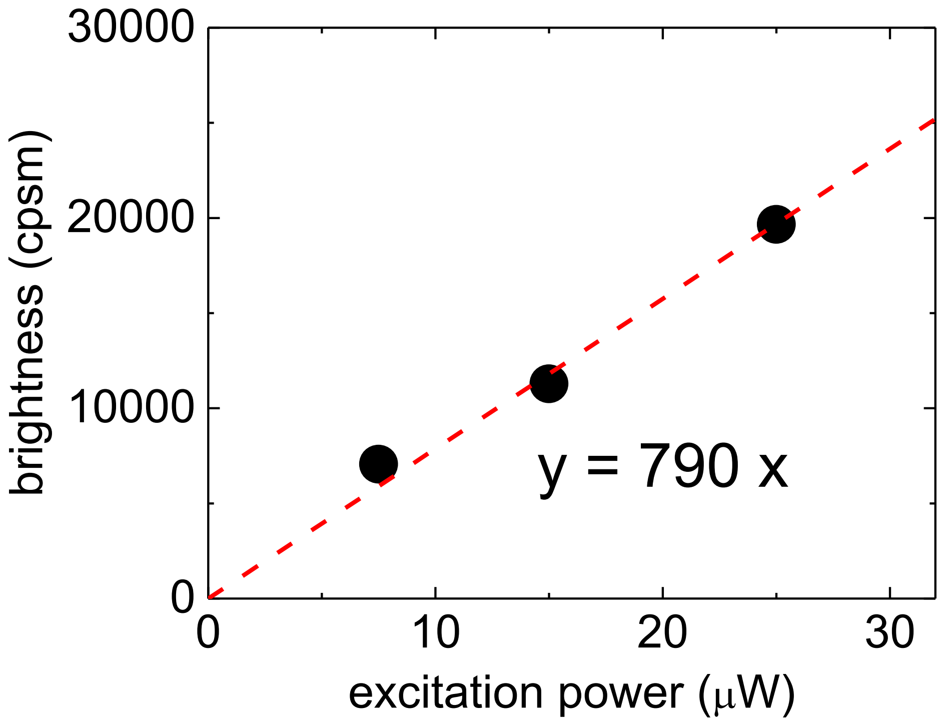

Supplementary Figure 9. Brightness of AcGFP1 in solution as a function of the excitation power entering the objective. The brightness is expressed in counts per second per molecule (cpsm). The red dashed line is a linear fit to the data with the intercept forced to 0.

Supplementary Note 1

Simplified formula for Raster Image Correlation Spectroscopy (RICS)

RICS analysis consists in computing the spatial ACF of an image or a series of images acquired in a raster-scan mode:

$$G(\xi,\eta)=\frac{1}{N_{frames}}\sum_{k=1}^{N_{frames}}\left(\frac{\langle I_k(x,y)I_k(x+\xi,y+\eta)\rangle}{\langle I_k(x,y)\rangle^2}-1\right)$$

Since the raster-scan image contains spatial and temporal information, related to the diffusion of the probe and the microscope scanning speed, the spatial correlation of the image also contains spatial and temporal information. The RICS ACF is then fitted to a proper diffusion model.
The RICS ACF shape depends on two contributions:

$$G(\xi,\eta)=S(\xi,\eta)\cdot G_{diffusion}(\xi,\eta)$$

Where $S(\xi,\eta)$ is the spatial component of the correlation function related to the laser scanning and is defined as:

$$S(\xi,\eta)=\exp\left(-\frac{\left[\left(\frac{|\xi|\delta x}{w_0}\right)^2+\left(\frac{|\eta|\delta y}{w_0}\right)^2\right]}{\left(1+\frac{4D(\tau_p|\xi|+\tau_l|\eta|)}{w_0^2}\right)}\right)$$

where δx and δy are the pixel sizes along the x and y scanning directions, respectively, τ_p is the pixel dwell time in x ($\tau_p=\delta x/v_x$ where v_x is the speed of the scanner along x), τ_l is the interline time in y, w_0 is the waist of the point spread function of the microscope.
The second term $G(\xi,\eta)$ is the spatial part related to the diffusion:

$$G_{diffusion}(\xi,\eta)=\frac{\gamma}{N}\left(1+\frac{4D(\tau_p|\xi|+\tau_l|\eta|)}{w_0^2}\right)^{-1}\times\left(1+\frac{4D(\tau_p|\xi|+\tau_l|\eta|)}{w_z^2}\right)^{-1/2}$$

where γ is the shape factor due to the distribution of the illumination in the focal volume (for a 3D Gaussian illumination γ=0.35) and where w_z is the waist along the z direction.
Considering only one spatial dimension (for instance the x-axis) the theoretical formula for the RICS ACF can be written as:

$$G(\xi) = S(\xi) \cdot G_{diffusion}(\xi)$$

where

$$S(\xi) = \exp\left(- \frac{\left(\frac{|\xi|\delta x}{w_0} \right)^2}{\left(1 + \frac{4D(\tau|\xi|)}{w_0^2} \right)} \right)$$

and

$$G_{diffusion}(\xi) = \frac{\gamma}{N}\left(1 + \frac{4D(\tau|\xi|)}{w_0^2} \right)^{-1} \times \left(1 + \frac{4D(\tau|\xi|)}{w_z^2} \right)^{-1/2}$$

where now τ is the pixel dwell time along that direction.
We now apply the substitution with the *sampling constants*:

$$\begin{cases} K_s = \dfrac{\delta x}{w_0} \\ K_t = \dfrac{4D\tau}{w_0^2} \end{cases}$$

Here K_s represents the spatial sampling of the point spread function (PSF) and K_t represents the temporal sampling, namely the ratio between the pixel dwell time and the diffusion time of the diffusing probe. As a matter of fact, since the diffusion time τ_D is defined as $\tau_D = \frac{w_0^2}{4D}$, it results $K_t = \frac{\tau}{\tau_D}$.

Now, the 1D ACF can be written as:

$$\mathrm{G}(\xi) = \mathrm{S}(\xi) \cdot \mathrm{G}_{\mathrm{diffusion}}(\xi) = \frac{\gamma}{N} \exp\left(-\frac{(|\xi|\mathrm{K_s})^2}{1 + \mathrm{K_t}} \right) \cdot (1 + \mathrm{K_t})^{-1} \cdot \left(1 + \frac{w_0^2}{w_z^2}\mathrm{K_t} \right)^{-1/2}$$

By doing this, we have made every contribution relative to the acquisition parameters and the probe diffusion implicit in the sampling constants. It's worth noticing that, since w_z/w_0 is a constant that depends on the PSF of the microscope (typically $w_z/w_0 \sim 3$ for a confocal PSF), the shape of $G(\xi)$ depends only on the sampling constants K_s and K_t.
