# Supplementary material for: Local raster image correlation spectroscopy generates high-resolution intracellular diffusion maps
Source: Commun Biol. 2018 Feb 8;1:10. doi: 10.1038/s42003-017-0010-6 (PMC6053083; doi:10.1038/s42003-017-0010-6)
Supplement: Supplementary file 2 — Description of Additional Supplementary Files [file 42003_2017_10_MOESM2_ESM.docx]

**Description of Additional Supplementary Files**

File Name: Supplementary Movie 1

Description: They are simulations of RICS data generated with the SimFCS software. They represent molecules diffusing in 3D with a diffusion constant D where D=4μm^2^

File Name: Supplementary Movie 2

Description: They are simulations of RICS data generated with the SimFCS software. They represent molecules diffusing in 3D with a diffusion constant D where D=10μm^2^

File Name: Supplementary Movie 3

Description: They are simulations of RICS data generated with the SimFCS software. They represent molecules diffusing in 3D with a diffusion constant D where D=16μm^2^

File Name: Supplementary Movie 4

Description: They are simulations of RICS data generated with the SimFCS software. They represent molecules diffusing in 3D with a diffusion constant D where D=20μm^2^

File Name: Supplementary Movie 5

Description: They are simulations of RICS data generated with the SimFCS software. They represent molecules diffusing in 3D with a diffusion constant D where D=24μm^2^

File Name: Supplementary Movie 6

Description: They are simulations of RICS data generated with the SimFCS software. They represent molecules diffusing in 3D with a diffusion constant D where D=26μm^2^

File Name: Supplementary Movie 7

Description: They are simulations of RICS data generated with the SimFCS software. They represent molecules diffusing in 3D with a diffusion constant D where D=30μm^2^

File Name: Supplementary Movie 8

Description: They are simulations of RICS data generated with the SimFCS software. They represent molecules diffusing in 3D with a diffusion constant D where D=36μm^2^

File Name: Supplementary Software 1

Description: The software runs under Matlab. The LRICS.m functions generates a diffusion map and a G(0) map from the analysis of an image stack (X-by-Y-by-T) or a carpet (X-by-T) dataset. A temporal moving average, a Gaussian smoothing and a custom selection of the dataset can be optionally selected.
